# *Vaccinium* as a comparative system for understanding of complex flavonoid accumulation profiles and regulation in fruit

**DOI:** 10.1093/plphys/kiad250

**Published:** 2023-05-02

**Authors:** Nick W Albert, Massimo Iorizzo, Molla F Mengist, Sara Montanari, Juan Zalapa, Andrew Maule, Patrick P Edger, Alan E Yocca, Adrian E Platts, Boas Pucker, Richard V Espley

**Affiliations:** New Cultivar Innovation, The New Zealand Institute for Plant and Food Research Limited (PFR), Palmerston North, 4472, New Zealand; Plants for Human Health Institute, North Carolina State University, Kannapolis, NC 28081, USA; Department of Horticultural Science, North Carolina State University, Raleigh, NC 27607, USA; Plants for Human Health Institute, North Carolina State University, Kannapolis, NC 28081, USA; Department of Horticultural Science, North Carolina State University, Raleigh, NC 27607, USA; New Cultivar Innovation, PFR, Motueka, 7198, New Zealand; USDA-ARS, Vegetable Crops Research Unit, Department of Horticulture, University of Wisconsin-Madison, Madison, WI 53706, USA; Plants for Human Health Institute, North Carolina State University, Kannapolis, NC 28081, USA; Department of Horticultural Science, North Carolina State University, Raleigh, NC 27607, USA; Department of Horticulture, Michigan State University, East Lansing, MI 48824, USA; MSU AgBioResearch, Michigan State University, East Lansing, MI 48824, USA; Department of Horticulture, Michigan State University, East Lansing, MI 48824, USA; HudsonAlpha Institute for Biotechnology, 601 Genome Way, Huntsville, 35806, USA; Department of Horticulture, Michigan State University, East Lansing, MI 48824, USA; HudsonAlpha Institute for Biotechnology, 601 Genome Way, Huntsville, 35806, USA; Institute of Plant Biology & BRICS, TU Braunschweig, Braunschweig, 38106, Germany; PFR, Auckland, 1142, New Zealand

## Abstract

The genus *Vaccinium* L. (Ericaceae) contains premium berryfruit crops, including blueberry, cranberry, bilberry, and lingonberry. Consumption of *Vaccinium* berries is strongly associated with various potential health benefits, many of which are attributed to the relatively high concentrations of flavonoids, including the anthocyanins that provide the attractive red and blue berry colors. Because these phytochemicals are increasingly appealing to consumers, they have become a crop breeding target. There has been substantial recent progress in *Vaccinium* genomics and genetics together with new functional data on the transcriptional regulation of flavonoids. This is helping to unravel the developmental control of flavonoids and identify genetic regions and genes that can be selected for to further improve *Vaccinium* crops and advance our understanding of flavonoid regulation and biosynthesis across a broader range of fruit crops. In this update we consider the recent progress in understanding flavonoid regulation in fruit crops, using *Vaccinium* as an example and highlighting the significant gains in both genomic tools and functional analysis.

**Advances**

- New *Vaccinium* genomic resources link genetic and physical maps
- Recent publication of blueberry, cranberry, and bilberry genome sequences
- Shared community resource hub at Genome Database for Vaccinium (www.vaccinium.org)
- New QTLs for anthocyanin content identified in blueberry and cranberry
- A network of MYB transcription factors has been identified to co-ordinately regulate flavonoid accumulation to help more broadly understand flavonoid control
- Increased data linking flavonoid pathway gene transcription with flavonoid chemistry, applicable to many crops

## Introduction

**Figure 1.**
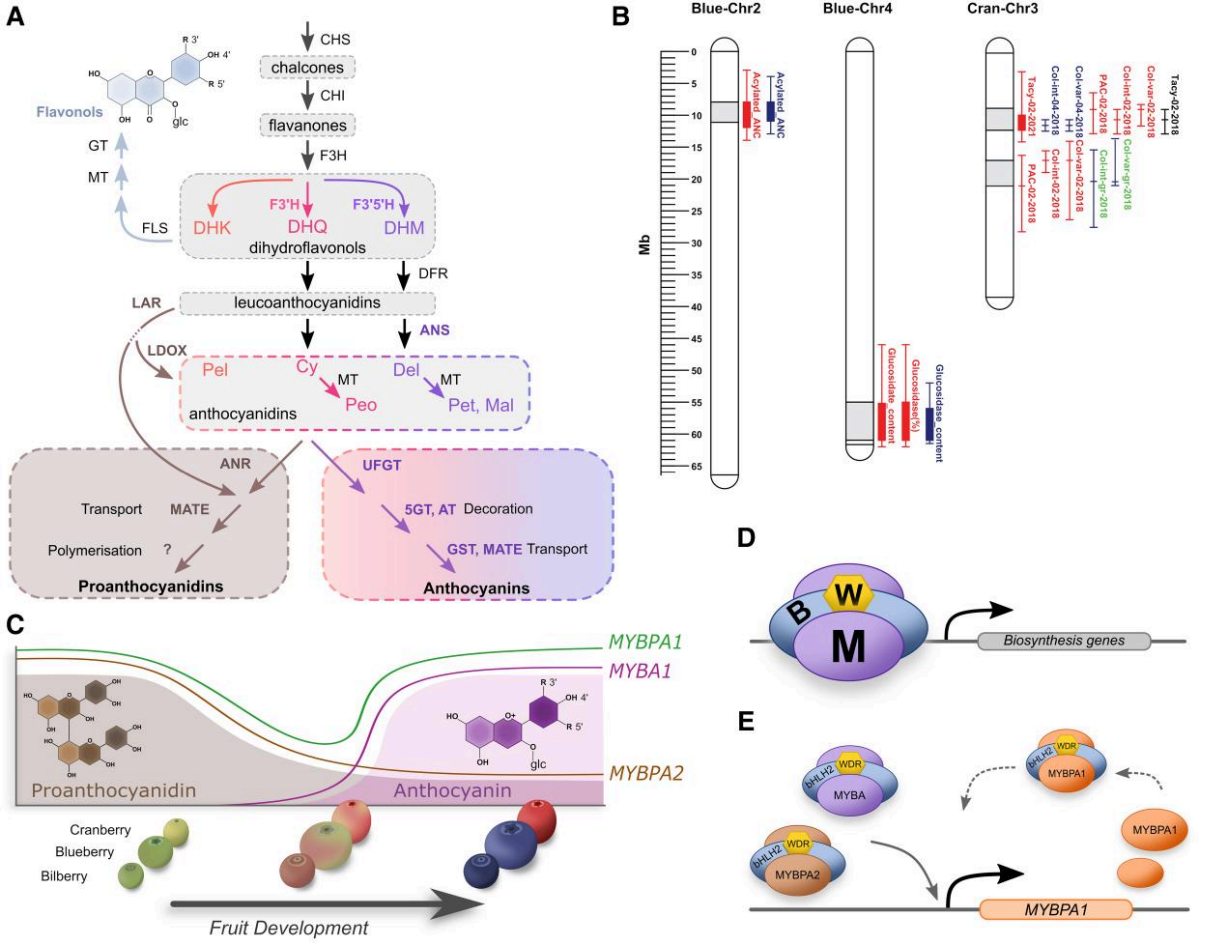
Biosynthesis, inheritance, and regulation of flavonoid traits in *Vaccinium.***A**) The biosynthetic pathway producing the major flavonoid classes reported in *Vaccinium* species: flavonols, PAs, and anthocyanins. Dihydroflavonols can vary in hydroxylation on the B-ring: dihydrokaemferol (DHK: 4ʹ), dihydroquercetin (DHQ: 3ʹ,4ʹ), and dihydromyricetin (DHM: 3ʹ,4ʹ,5ʹ). The hydroxylation pattern is retained by subsequent compounds in the pathway, corresponding to the 3 major anthocyanidins pelargonidin (Pel), cyanidin (Cy), and delphinidin (Del), and methylated derivatives peonidin (Peo), petunidin (Pet), and malvidin (Mal). LDOX and ANS can refer to the same gene, although some species, including *Vaccinium*, possess distinct *LDOX* and *ANS* genes. **B**) Anchored QTLs for flavonoids and color detected in blueberry and cranberry. Genomic QTL locations for anthocyanin acylation and glycosylation mapped in blueberry chr2 and chr4, respectively, across 2 mapping populations. QTL regions in blue represent those mapped in NxHP (Montanari et al. 2022), and QTLs in red are those mapped in DSxJ (Mengist et al. 2022b). Markers spanning 2-LOD intervals were used to anchor these QTLs. The sections in a grey shaded box indicate the genomic regions in chr2 (chr2: 8,005,747–11,180,545 bp) and chr4 (chr4: 55,208,167–60,978,559) where the regions spanning 2-LOD intervals for the same trait (e.g. acylated anthocyanin) overlap across the 2 mapping populations/studies. Genomic locations of QTLs for TAcy, PAC, color variation, and color intensity mapped in cranberry across 3 mapping populations and 2 genetic studies (Diaz-Garcia et al. 2018, 2021). QTL regions in blue represent those mapped in CNJ04–02, QTLs in red represent QTLs mapped in CNJ02–01, and QTLs in green represent QTLs mapped in GRYG. Markers spanning the 1.5-LOD interval were used to anchor these QTLs. The grey box sections indicate the genomic regions in chr3 (chr3: 8,700,846–12,176,781 bp, and chr3:16,922,077–20,907,119), where the region spanning the 1.5-LOD intervals for the same or related traits (e.g. TAcy and color intensity) overlap across the populations/studies. **C**) Flavonoid profiles change during bilberry, blueberry, and cranberry fruit development. The shift from PAs during early, immature stages are linked with SG5/MYBPA2 genes, and anthocyanin accumulation at ripening is linked with SG6/MYBA genes. MYBPA1 has a biphasic expression profile, correlating with both PAs and anthocyanins. **D**) Anthocyanins and PAs are regulated by MBW TF complexes, which activate flavonoid biosynthetic gene expression. The target genes regulated by MBW complexes are primarily determined by the type of R2R3-MYB. The exact stoichiometry is unknown, but MBW complexes can contain 2 MYBs via dimerized bHLH proteins. **E**) MBW complexes containing MYBA and MYBPA2 proteins directly regulate *MYBPA1* genes, which may then be reinforced by MYBPA1 itself.

Flavonoid biosynthesis is one of the best characterized specialized metabolite pathways in plants, owing to a long history of research on mutants that are phenotypically conspicuous. Examples include petunias (*Petunia hybrida*) and snapdragons (*Antirrhinum majus*) with altered flower colors, maize (*Zea mays*) with altered cob colors, and Arabidopsis with pale seed coats (transparent testa/tt mutants). Studies from these and other systems have helped build an understanding of the flavonoid biosynthetic pathway (Fig. 1A; (reviewed in Tohge et al. 2017; Albert et al. 2022). However, we are still learning about the accumulation of flavonoids; as they increasingly become a breeding target for nutritional enhancement in crops, so the need for genetic markers becomes more important. Berry crops, such as blueberry, often contain high concentrations of diverse flavonoids, making them useful to study the biosynthesis and control of flavonoids. The recent generation of *Vaccinium* genome assemblies, transcriptome assemblies, and metabolomes opened the opportunity to study flavonoid production in a range of fruit crops.

The genus *Vaccinium* L. (Ericaceae) comprises approximately 450 diverse species, including the following major commercial crops: northern highbush blueberry (*V. corymbosum* L.), rabbiteye blueberry (*V. virgatum, formerly V. ashei*), lowbush blueberry (*V. angustifolium*), bilberry (*V. myrtillus* L.), cranberry (*V. macrocarpon*), and lingonberry (*V. vitis-idaea* L.) (Luby et al. 1991; Retamales and Hancock 2018). The berries of many wild *Vaccinium* species were traditionally used as foods and medicines; however, they remained undomesticated until the 20th century, when selection and breeding efforts began (Ehlenfeldt 2008; Vorsa and Zalapa 2019). There is now an increasingly sophisticated approach to *Vaccinium* crop improvement (Edger et al. 2022). *Vaccinium* ploidy spans diploid (2×), tetraploid (4×), and hexaploid (6×) species, although their ancestry and taxonomy are not yet fully resolved (Lyrene et al. 2003). Recent progress developing genetic resources for *Vaccinium* includes genome assemblies for blueberry, bilberry, and cranberry (Colle et al. 2019; Diaz-Garcia et al. 2021; Wu et al. 2021; Mengist et al. 2022a ) and a centralized genomics hub for genomic and transcriptomic sequences: Genome Database for Vaccinium (www.vaccinium.org).

Global production and consumption of blueberries and other *Vaccinium* berries is growing, benefiting from an increasing body of research demonstrating their potential health promotion (Norberto et al. 2013). This translates to consumer behaviors, with health benefits being a key driver for blueberry-purchasing decisions (Gilbert et al. 2014). The health benefits of *Vaccinium* berries are attributed to their phytochemical content, including various flavonoid and other phenolic compounds. For instance, *Vaccinium* berry consumption has been associated with numerous health benefits: alleviation of inflammation, fever, digestive and urinary tract disorders; acting as an antiseptic (Tundis et al. 2021); association with a reduced risk of cardiovascular disease, some cancers, and type 2 diabetes; and benefits to neuroprotection, cognition, and vision (Ma et al. 2018; Kalt et al. 2020). Although much of this research has focused on blueberry and cranberry (Nemzer et al. 2022), there is also increasing evidence for the health benefits of other *Vaccinium* species, including bilberry (Sharma and Lee 2022) and lingonberry (Kowalska 2021).

Flavonoids have diverse physiological roles in planta (reviewed in Albert et al. 2022). Flavonols provide protection against UV-B radiation and abiotic and biotic stresses and have different roles according to tissue type and developmental stage. In vegetative tissues, anthocyanins help to screen excess light in stressed tissues and possibly act as antioxidants, protecting cells against reactive oxygen species–induced oxidative damage. Anthocyanins perform important roles for animal signaling, providing visual cues to attract pollinators and coloring fruit upon ripening to attract seed distributers. Another class of flavonoids, proanthocyanidins (PAs), are astringent compounds that deter herbivory. They are abundant in *Vaccinium* vegetative tissues (Hokkanen et al. 2009) but also important in fruit by deterring consumption before fruit (and seed) maturation. *Vaccinium* berries contain high concentrations of anthocyanins, which are generally restricted to the fruit skin, but in some species, like bilberry, they can also be present in the flesh (Riihinen et al. 2008). The anthocyanins in *Vaccinium* include various glycosides of cyanidin, delphinidin, malvidin, peonidin, and petunidin, and the concentrations and moiety decorations can vary within and between species (Kalt et al. 1999; Veberic et al. 2015). Flavonoid production appears spatially orchestrated within the berry as it develops, with the specialized metabolism of flavonoids following a pattern as the fruit matures (Dare et al. 2022). This suggests synchronized transcriptional control of the pathway. Although there is good evidence that anthocyanin production is developmentally coordinated, the type and concentration can vary considerably with geographic location and environmental conditions (Zoratti et al. 2015; Karppinen et al. 2016).

*Vaccinium* berries also contain an impressive variety of other flavonoids and other notable phenylpropanoids. In blueberry, these are largely hydroxycinnamic acids, stilbenes, PAs, and flavonols, all being at their highest abundance early in fruit development and decreasing in concentration as anthocyanin production rises (Dare et al. 2022). It is quite possible that a variety of phytochemicals contributes to the overall bioactivity of *Vaccinium* berries. For example, Carey et al. (2013) showed that blueberry extract had greater efficacy than pure anthocyanin or stilbenes in protecting against stress-induced inflammation. They suggested synergistic effects between anthocyanins, stilbenes, and other secondary metabolites present in blueberries may be responsible for the increased efficacy of extracts compared with pure compounds. Indeed, synergistic effects of anthocyanin and stilbenes have been demonstrated for an inflammatory bowel disease model, using transgenic tomato extracts producing anthocyanins, flavonols, and stilbenes, or all of these, showing significant improvement in reversing disease. This approach allows the contribution of different classes of phenolic metabolites to be assessed in different combinations in a well-controlled manner. Similar effects were also observed comparing white and red grape skins, which contain different quantities of anthocyanins and stilbenes (Scarano et al. 2017).

This review distils recent progress in understanding flavonoid regulation using *Vaccinium* as an example of where recent significant gains in both genomic tools and functional analysis have been made. There is strong momentum within the *Vaccinium* research community to further progress to drive increasingly sophisticated breeding approaches but also to develop knowledge that will helpus understand flavonoid regulation in other fruit crops.

## Flavonoid biosynthesis and chemical diversity in *Vaccinium*

Most flavonoids are 15-carbon structures comprised of 3 rings called the A-, B-, and C-rings. The first committed step toward flavonoid biosynthesis is catalyzed by chalcone synthase (CHS), often encoded by multigene families of differentially regulated genes. Although conversion of naringenin chalcone into naringenin occurs spontaneously in vitro, this reaction is catalyzed by chalcone isomerase (CHI) in planta. The flavonoid backbone is then hydroxylated by flavanone 3-hydroxylase (F3H) and may be hydroxylated further by flavonoid 3ʹhydroxylase (F3ʹH) and flavonoid 3ʹ5ʹhydroxylase (F3ʹ5ʹH). The 3 resulting dihydroflavonols, which differ in the number of hydroxyls on the B-ring, progress into the pathway toward branches for flavonols or anthocyanins/PAs. The metabolic flux into the 2 competing branches depends on substrate preferences of dihydroflavonol 4-reductase (DFR) and flavonol synthase (FLS). In bilberry, flavonols accumulate from early stages of fruit development, primarily in the skin (Dare et al. 2022). A similar pattern was observed in strawberry (*Fragaria vesca*), where flavonols dominate during early developmental stages of fruits, whereas anthocyanin accumulate at late stages (Miosic et al. 2014). In blueberry and bilberry fruit, these are primarily quercetin glycosides with traces of myricetin glycosides (or methylated derivatives), although kaempferol-based flavonols are detected in leaves (Riihinen et al. 2008). *FLS* genes were assigned to 3 lineages in the *Vaccinium* species. One lineage shows perfect conservation of all amino acids that have been reported to be functionally important (Pucker et al. 2020). The other 2 lineages differ at position S225 and E295 from the Arabidopsis FLS1, respectively. It is possible that one of these substitutions altered the substrate specificity of FLS. Spatio-temporal differences in gene expression of these *FLS* genes could explain the flavonol pattern observed in leaves and fruits. Methylated derivatives of quercetin and myricetin are also produced, but this occurs later in fruit development when anthocyanin biosynthesis occurs, coinciding with flavonoid *O*-methyl transferase expression (Günther et al. 2020). Cranberry has a different flavonol profile, with significant concentrations of myricetin-glycosides in addition to quercetin-glycosides (Vvedenskaya et al. 2004; Singh et al. 2009).

Flavonoid biosynthesis genes are generally conserved across plant species (Supplemental Table S1). Differences affect the flavone biosynthesis, which relies on the P450 cytochrome flavone synthase II (FNSII) in most plant species. However, no FNSII ortholog was discovered in Arabidopsis, kiwifruit (*Actinidia chinensis)*, cranberry, or bilberry. Only species of the Apiaceae are known to produce flavones through the F3H-derived flavone synthase I. Another important difference between species is the hydroxylation pattern of anthocyanins. F3′5'H is required for the formation of delphinidins and the resulting blue pigmentation. The F3′5'H enzyme functionality evolved multiple times independently from the F3′H lineage. Likely functional F3′5'H candidates were discovered in grape (*Vitis vinifera*), kiwifruit, and the 3 *Vaccinium* species. This functionality is missing in Arabidopsis, apple (*Malus domestica*), and strawberry. Arabidopsis does not harbor leucoanthocyanidin reductase (LAR), which leads to lower diversity of PAs compared with other species. Orthologs of 3AT and 3MAT were not detected in most of the species, which suggests that these might be lineage-specific functions. A parallel or convergent evolution of this functionality remains possible. Copy number differences between species seem to be tandem duplications that form gene arrays.

The anthocyanin color is primarily determined by the hydroxylation state of the B-ring: pelargonidin/orange-red (4ʹ), cyanidin/red-magenta (3ʹ4ʹ), and delphinidin/purple-blue (3ʹ4ʹ5ʹ). Pelargonidin-based anthocyanins are not reported in *Vaccinium* species, suggesting that, as observed in other species (Johnson et al. 2001), DFR has strong substrate preference for dihydroquercetin (3ʹ4ʹ) or dihydromyricetin (3ʹ4ʹ5ʹ) and not dihydrokaemferol (4ʹ). However, an inspection of the DFR sequences in the *Vaccinium* species revealed N at the critical position corresponding to 134 in the reference sequence analyzed by Johnson et al. 2001, which suggests that these enzymes should be able to accept all 3 dihydroflavonols. No DFR sequence with the dihydrokaemperol excluding D at this position was detected. It is possible that additional amino acid residues are restricting the accepted substrate or that a strong hydroxylase activity restricts the available substrate to dihydroquercetin and dihydromyrecitin.

Blueberries and bilberries have complex anthocyanin profiles consisting of cyanidin- and delphinidin-based anthocyanins (and methylated forms: peonidin, malvidin, petunidin) compared with red-fruited cranberries, lingonberries, and some other wild *Vaccinium* species, which produce cyanidin/peonidin. The lack of delphinidin-based anthocyanins in these red-fruited species could be due to the absence of F3ʹ5ʹH activity. This is similar to apple and strawberry, which do not have F3′5′H activity due to a lack of the corresponding gene (Han et al. 2010). Differences regarding the F3′5′H activity have also been reported for grape cultivars and are also largely due to transcriptional regulation (Falginella et al. 2010). However, cranberry is reported to produce trihydroxylated flavonols (myricetin) in fruit (Vvedenskaya et al. 2004), and the cranberry genome has an intact *F3ʹ5ʹH* gene (vmacro08261-RA) as well as other apparently pseudogenized copies. Thus, the lack of delphinidin may be because the timing of F3ʹ5ʹH activity does not coincide with the expression of other anthocyanin biosynthesis genes. Multiple genes encode F3ʹ5ʹH in blueberries, but it is unclear if these are differentially regulated for producing different flavonoid classes (e.g. flavonols vs anthocyanins).

Formation of anthocyanidins, common to anthocyanin and PA pathways, is catalyzed by anthocyanidin synthase (ANS) or leucoanthocyanidin dioxygenase (LDOX). In some species these refer to a single gene (e.g. Arabidopsis), but in others, including blueberry (Lafferty et al. 2022a), they can be phylogenetically separated into distinct clades, with *ANS* genes regulated for anthocyanin production and *LDOX* genes for PAs (Jun et al. 2018). Glycosylation of the anthocyanidin, e.g. by UDP-glucose:flavonoid-3-*O*-glucosyltransferase (UFGT), forms anthocyanin pigments. In blueberries and cranberry, the major flavonoid glycosylation patterns are typically galactosides and arabinosides, with lower concentrations of glucosides (Prior et al. 2001). In contrast, *V. oxycoccus* (small cranberry) contains a greater proportion of glucosides than galactosides (Vorsa and Polashock 2005), suggesting a great variety of anthocyanin decorations may exist within nondomesticated *Vaccinium* species. Additional modifications/decorations of the anthocyanin molecules can generate even greater diversity of anthocyanin structures through glycosylation in other positions (5GT) or acylation (acyl transferase). Anthocyanins with 3-glucose 6-acetyl modifications are found in some highbush blueberries cultivars (e.g. “Nui”), but in other *Vaccinium* taxa these are present at low concentrations (e.g. lowbush blueberry) or not detectable (rabbiteye blueberry, cranberry) (Prior et al. 2001; Günther et al. 2020). These anthocyanin modifications alter solubility, stability, and the ability of anthocyanins to form supra-molecular complexes with other flavonoids and phenolics (e.g. co-pigmentation with flavonols). They also alter bioaccessibility and bioavailability when consumed (He et al. 2005; Mengist et al. 2020).

After synthesis at the endoplasmatic reticulum, anthocyanins are transported to the central vacuole for long-term storage. Two different models for anthocyanin transport have been proposed: (1) vesicle-mediated or (2) direct transport through the cytoplasm and across the tonoplast (reviewed by Pucker and Selmar 2022). The latter is thought to require the activity of a specific glutathione-*S-*transferase and additional transporters [e.g. including multi-drug and toxin extrusion transporter (Zhao et al. 2011) and ATP-binding cassette subfamily C transporter (Goodman et al. 2004)]. The transport of anthocyanins is poorly understood and requires additional research.

For PA biosynthesis, leucoanthocyanidins can be converted into (+)-catechin by LAR, which can either go on to polymerize into PAs or, as demonstrated by Jun *et al.* (2018), be converted into anthocyanidin (e.g. Cy) by LDOX. Anthocyanidins are then reduced by anthocyanidin reductase (ANR) into (−)-epicatechin. Blueberry and other *Vaccinium* species have both *ANS* and *LDOX* genes, which are differentially expressed during anthocyanin or PA biosynthesis, respectively (Lafferty et al. 2022b). This not only provides redundant activity, but it may also contribute to metabolite complexity, as proposed for *Medicago truncatula* by Jun et al. (2018). The PA monomers can react to form oligomers and polymers, forming PAs, although this aspect is not fully understood. Transport to the vacuole requires specific proton pumps (e.g. AtTT13) and MATE transporters (Zhao and Dixon 2009; Appelhagen et al. 2015).

*Vaccinium* fruit tend to have complex flavonoid profiles. This includes mixtures of methylated flavonoids and varying types of glycosylation and acylation. Some of the genes responsible for modifying and decorating the flavonoids are not yet identified. Flavonoid glycosyl transferases and other decorating enzymes belong to very large gene families, and their functionality is difficult to infer from sequences (Yonekura-Sakakibara and Saito 2014; Bontpart et al. 2018). Flavonoids are not the only bioactive compounds in *Vaccinium* fruit, and it is likely synergistic actions with other phenolics [e.g. chlorogenic acid (CHA) and stilbenes] occur to confer health benefits when consumed. Quantitative genetics can make a significant contribution to developing novel crops with improved health benefits by identifying loci associated with particular flavonoid species and phenolic compounds to assist breeding efforts.

## Flavonoid genetics in blueberry and cranberry

With the recent development/adoption of advanced genotyping platforms and genomic resources in *Vaccinium*, improvements in understanding the genetic mechanisms controlling flavonoid accumulation have been made for blueberry and cranberry. In this review, we summarize data from available flavonoid studies in these 2 important crops, emphasizing quantitative trait loci (QTLs) that were stable across years and/or detected across multiple genetic backgrounds, and we anchor them to the physical map. Regions spanning these QTLs are the most likely targets for studies on flavonoid genetics in these crops.

### Anthocyanin QTLs in blueberry

For blueberries, 2 studies focused on dissecting the genetic mechanism controlling CHA content and anthocyanin content and composition (Mengist et al. 2022b; Montanari et al. 2022).

Mengist et al. (2022b) used a mapping population (DSxJ), representing highbush blueberry cultivars “Draper” and “Jewel,” and identified 180 QTLs for total and individual anthocyanin content, relative anthocyanin composition, and CHA (Supplemental Table S2). The study highlighted identification of QTLs for CHA, total anthocyanin content, and the conjugations of anthocyanin with the different sugar moieties and acylation. These QTLs were stable across the years and explained a large fraction (up to 80%) of the phenotypic variation. QTLs for CHA and anthocyanin acylation overlapped in the same region of chr2, and haplotype analysis indicated that these QTLs were located in different haplotypes, which implies they could be independently selected. Montanari et al. (2022) used a different mapping population (NxHB) representing 2 different highbush cultivars, “Nui” and “Hortblue Petite.” Consistent with the results of Mengist et al. (2022b), they mapped stable and strong effect QTLs on chr2 and chr4 that regulated the concentration of acylated anthocyanins and glucoside-based anthocyanins, respectively.

In this review, we anchored the 2 clusters of QTLs detected on chrs 2 and 4 in the 2 studies to the W85 v2 genome (Mengist et al. 2022b), showing that they overlap (Fig. 1B). The QTLs controlling acylation on chr 2 span a common 3.2-Mb-long region between positions 8.0 and 11.2 Mb, and the QTLs linked to glycosylation mapped to chr4 overlap over a 4.8-2Mb-long region between positions 56.2 and 61.0 Mb (Fig. 1B). Co-mapping of these QTLs across different genetic backgrounds represents the first level of QTL validation. Initial efforts to mine these regions for candidate genes by Mengist et al. (2022b) and Montanari et al. (2022) identified a putative *UDP-glucosyl transferase* gene (Vcev1_p0.Chr04.11988), 2 putative *DFR* genes (Vcev1_p0.Chr4.11916, Vcev1_p0.Chr4.11918), and a *MYB* transcription factor (TF) (Vcev1_p0.Chr4.11990) as candidate underpinning the chr4 QTL. Two *BADH acyltransferases* genes (Vcev1_p0.Chr02.03371 and Vcev1_p0.Chr02.03383), a *MYB* TF (Vcev1_p0.Chr2.03364), and a *DFR* gene (Vcev1_p0.Chr2.03323), were identified as candidates underlying the chr2 QTL. Candidacy of the 2 *BADH-acyltransferase* genes in chr2 and the *UDP-glucosyl transferase* in chr4 was also supported by gene expression analysis by Mengist et al. (2022b).

### Anthocyanin and proanthocyanin QTLs in cranberry

To date, only one study has examined the genetics of flavonoid content in cranberry (Diaz-Garcia et al. 2018). This focussed on total anthocyanin content (TAcy) and PA content (PAC), evaluated using spectrophotometric methods, with fruit color intensity and variation measured by digital image analysis. QTL analysis was performed using an integrated map representing 3 mapping populations (CNJ02–01, CNJ04–02, and GRYG) that enabled direct comparison of the locations of the QTLs detected across the 3 families (Supplemental Table S3). Several QTLs for TAcy and/or PAC overlapped with QTLs for color variation and/or intensity, which indicated that these traits are correlated (Diaz-Garcia et al. 2018). Seven regions in chrs 1, 3, 6, 9, and 12 harbored QTLs associated with the same/correlated traits (e.g. TAcy and color intensity) across 2 or 3 populations, suggesting a stable effect across genetic backgrounds. However, only 2 of these 7 regions contained QTLs that explained a considerable proportion (>5%) of phenotypic variation. In particular, one region on chr3 of the GRYG and CNJ02–01 populations harbored overlapping QTLs for color intensity, color variation, and PAC that explained up to 19% of phenotypic variation. The second hotspot was also identified on chr3 in the CNJ02–01 and CNJ04–02 populations, and it included QTLs for all the traits (PAC, TAcy, color variation, color intensity) that explained up to 39.5% of the phenotypic variation. In 2021, Diaz-Garcia et al. (2021) reanalyzed the same TAcy data using an improved linkage map of population CNJ02–01 and confirmed a strong effect of QTL in the same chr3 region that explained up to 51.2% of phenotypic variance. Here, anchoring of the overlapping QTLs on chr3 to the “Stevens’ genome identified 2 regions spanning 3.5 and 4 Mb. Within the first region, located between 8.7 and 12.2 Mb on chr 3, they identified a cluster of 3 R2R3 MYB110 TF genes (vmacro18045:10437125-10439365, vmacro18044:10493992-10499550, and vmacro18043:10561041-10564609) as candidate for the control of TAcy in cranberry. Two of these genes, vmacro18044 and vmacro18045, exhibited extremely high similarity with blueberry *MYBA* TF, which is known to directly activate anthocyanin biosynthesis (Plunkett et al. 2018). A third unannotated *MYBA1* gene was identified in cranberry within this locus (Wu et al. 2021). In the second region, which spanned position 16.9–20.9 Mb on chr 3, a *DFR* gene was annotated as a potential candidate underlying this QTL.

Additionally, an analysis of the anthocyanin content in a diverse cranberry germplasm suggested that genetic diversity for a range of anthocyanin traits exists. This includes variation in the content of cyanidin and its methylated derivative, peonidin, and variation in glycosylation (galactosides/arabinosides/glucosides) (Vorsa et al. 2003). QTLs associated with these traits have not yet been reported for cranberry.

The studies reported here for blueberry and cranberry add to a list of works on the genetic control of flavonoid content in fruit crops, mostly in species belonging to the family Rosaceae, such as strawberry (Labadie et al. 2020, Pott et al. 2020), peach (*Prunus persica*; Abdelghafar et al. 2020), Asian plum (*Prunus salicina*; Valderrama-Soto et al. 2021; Battistoni et al. 2022), sweet cherry (*Prunus avium*; Calle et al. 2021), and apple (Kumar et al. 2022). Such studies probably have been boosted by the recent improvements in flavonoid identification and quantification methodologies and the increased interest around their beneficial health properties. All these studies reported on the quantitative nature of flavonoid content and highlighted how this trait is affected by the environment. Nevertheless, major QTLs and chromosome hotspots were detected in all species and were stable across seasons/environments, suggesting that a small number of genes have a strong effect on the accumulation of flavonoids. This is important because it opens the door to the application of marker-assisted selection for flavonoid content in fruit crops.

Of all the different classes of flavonoids, anthocyanins have received the greatest attention in fruit crops. However, most studies investigated the genetic control of anthocyanin content but not their composition. On the contrary, both Mengist et al. (2022b) and Montanari et al. (2022) showed that most genetic variation for blueberry anthocyanin is associated with glycosylation and acylation of the molecule, and genetic contribution for total anthocyanin is limited relative to their composition. An analogous study performed by Sun et al. (2020) in grape found QTLs linked to both the methylation and acylation of the anthocyanin molecules in the skin of grape berries. Analyzing the specific composition of anthocyanins in other fruit crops, including cranberry, is therefore important to fully understand the genetic regulation of these compounds.

In all these fruit crops, including blueberry and cranberry, genes encoding MYB TFs were identified in the major QTL regions, confirming their well-known role in anthocyanin regulation. However, other candidate genes that appeared of particular interest in blueberry are the *BADH-acyltransferases* and the *UDP-glucosyl transferases*, which have only been reported in peach (Abdelghafar et al. 2020) and *Citrus* (Mou et al. 2021). It is, however, important to note that genes involved in anthocyanin acylation and glycosylation belong to very large gene families, for which the function is not well conserved across species, making them difficult to identify. Continuing to leverage genetic studies is likely to be the most effective way to identify these gene.

Because work on flavonoid genetics in *Vaccinium* is still in its relative infancy, the next obvious step would be to expand association analyses to flavonoid classes other than anthocyanins. Looking at the variation of a large number of flavonoid compounds and their biochemical relationships can provide insights into their regulatory gene networks and contribute to a better understanding of their biosynthetic pathways, as was shown in strawberry by Pott et al. (2020).

## Flavonoid regulation

The regulation of anthocyanin and proanthocyanin biosynthesis is well characterized. The flavonoid biosynthesis genes are transcriptionally regulated, and flavonoids accumulate in the tissues where coordinated expression of the biosynthesis and transport genes for a particular metabolite occurs. This is mediated by TFs, particularly those of the MYB family. The R2R3-MYB genes can be phylogenetically clustered into subgroups (SG), which generally have conserved functions among flowering plants; e.g. those belonging to SG5 regulate PAs, SG6 regulates anthocyanins, and SG7 regulates flavonols or flavones (Stracke et al. 2001; Albert and Allan 2021). These insights have largely come from genetic models such as maize, petunia, Antirrhinum, and Arabidopsis (reviewed in Albert et al. 2022), but it is becoming clear flavonoid regulation is more nuanced, particularly in crops with complex flavonoid profiles that change throughout different developmental stages (Fig. 1C).

### The MYB-bHLH-WDR complex

In addition to pathway-specific MYBs, basic helix-loop-helix (bHLH) and WD-repeat (WDR) TFs are essential for regulating anthocyanins and PAs. These TFs can form MYB-bHLH-WDR (MBW) complexes (Fig. 1D), which bind to the promoters of target biosynthesis genes, activating transcription and ultimately resulting in metabolite accumulation (Baudry et al. 2004; Albert et al. 2014; Xu et al. 2015). The bHLHs involved belong to SG IIIf but can be further separated into bHLH-1 and bHLH-2 subtypes, corresponding to AtEGL3/PhJAF13/AmDel and AtTT8/PhAN1/AmIncI, respectively (Feller et al. 2011; Albert et al. 2021). These proteins share some activity but also have nonredundant functions, varying between species; for example, TT8 (bHLH-2) is essential for PA regulation in Arabidopsis seed coats but has redundant activity for anthocyanin regulation with the bHLH-1 proteins (EGL3 and GL3) (Nesi et al. 2000; Baudry et al. 2006; Gonzalez et al. 2008). In petunia and other Solanaceae species, *AN1* is essential for anthocyanin regulation, vacuolar acidification, and seed coat formation (presumably PAs) despite expressing the functional bHLH1 gene, *JAF13* (Montefiori et al. 2015; Spelt et al. 2000, 2002). Hierarchical regulation of bHLH2 genes by MBW complexes is now well established in anthocyanin and PA regulation and may be initiated by MBW complexes containing bHLH-1 proteins (Baudry et al. 2006; Albert et al. 2014); it is conserved in *Vaccinium* (Lafferty et al. 2022b). This provides a feed-forward mechanism to enhance responses by ensuring sufficient abundance of key proteins and can also expand the expression domain of bHLH2 genes into additional tissues (Albert et al. 2021). Other conserved aspects of MBW activity, such as repression by SG4 MYB and R3-MYB proteins (Albert et al. 2014), have been identified and characterized in *Vaccinium* species for anthocyanin and PA regulation (Lafferty et al. 2022b). Given the shared roles for WDR and bHLH components within MBW complexes, it is primarily the R2R3-MYB (activator) that determines the specificity of the genes and pathways regulated.

### Flavonol regulation

Flavonols help protect plants against abiotic stress, acting as UV-B sunscreens and scavengers of reactive oxygen species. Consequently, their biosynthesis is typically promoted by light (including UVA and UVB) in vegetative tissues, immature flowers, or developing fruit, particularly in exposed skin tissues. Their production is regulated by SG7 MYBs, such as AtMYB12 in Arabidopsis and VvMYBF1 in grape (Mehrtens et al. 2005; Czemmel et al. 2009), but also by bZIP TFs like HY5 (Hartmann et al. 2005), functioning independently of MBW complexes. These directly activate the promoters of genes necessary for flavonol production (*CHS*, *CHI*, *F3H*, and *FLS*). SG7 MYBs have been identified and characterized in numerous fruit crops, including apple (*MsMYB22;*Wang et al. 2017), pear (*Pyrus bretschneideri)PbMYB112b;*Zhai et al. 2019), strawberry (Martínez-Rivas et al. 2023), and recently *VmMYBF* in bilberry (Karppinen et al. 2021). Genetic variants of these regulators can confer quantitative variation in flavonol content, such as pink-fruited tomato (*yellow* locus: *Slmyb12*; Ballester et al. 2010), pepper (*Capsicum annum: CaMYB12-like*; Wu et al. 2023), and pear (Zhai et al. 2019). There has been recent progress in understanding flavonol regulation, but, given the wide variation in flavonol concentration and the influence of environment, it is likely that there are other regulatory pathways and genes yet to be discovered.

### Anthocyanin regulation

The SG6 MYBs are central for regulating anthocyanins, forming transcription factor complexes with bHLH and WDR proteins (MBW) that activate genes for anthocyanin biosynthesis and transport. These genes often exist in small gene families, with distinct expression profiles, and it is common to have genes linked with vegetative pigmentation (stress/light responsive) and those with more specific roles in flower pigmentation or fruit color (Schwinn et al. 2006; Albert et al. 2011; Matus et al. 2017). Although initially characterized in model plant species, significant insights have also come from fruit crops. This is because variants of SG6 MYB genes can give rise to fruit with different colors or patterns, which have been selected for or against. Thus, SG6 MYB alleles have been identified in numerous crops that are responsible for determining anthocyanin content or patterning (Paauw et al. 2019; reviewed in Albert and Allan 2021).

Despite the well-established role of SG6 MYBs in controlling anthocyanins, these remained elusive for some time in *Vaccinium* species compared with other fruit species. Early studies identified *MYBPA1* genes as the candidate primary regulator rather than a typical SG6 MYB, based on expression patterns linked with pigmentation. For example, silencing a bilberry homologue of the MADS-box ripening regulator *FRUITFUL* resulted in a loss of *MYBPA1* expression and anthocyanins (Jaakola et al. 2010). Similarly, albino mutants of bilberry and bog bilberry (*V. uligonosum*) showed loss of *MYBPA1* expression during berry ripening (Primetta et al. 2015; Zorenc et al. 2017). The MYBPA1-type proteins belong to a distinct subgroup of R2R3-MYB originally identified in grape (VvMYBPA1) and absent from Arabidopsis. *VvMYBPA1* was initially considered a PA regulator because it could activate the expression of the PA-biosynthesis genes *ANR* and *LAR* required to make catechin and epicatechin monomers, and it could complement the Arabidopsis *tt2* mutant (Bogs et al. 2007). *VvMYBPA1* expression peaks early during fruit development when PA biosynthesis occurs and again at véraison in the skin, where anthocyanin accumulation occurs (Bogs et al. 2007). Yet VvMYBPA1 was unable to activate *UFGT* (Bogs et al. 2007), a key step for anthocyanin biosynthesis in grape that is regulated by the VvMYBA proteins (SG6) (Walker et al. 2007; Matus et al. 2017). Similar temporal patterns of metabolite accumulation are shared between grape and blueberry/bilberry (Czemmel et al. 2012; Primetta et al. 2015; Karppinen et al. 2016) (Fig. 1B), suggesting that both *MYBPA1* and SG6/*MYBA* genes may be necessary for activating anthocyanin production in these species, in contrast to simpler model species.

The SG6 MYB genes were identified in blueberry by taking a targeted approach. *VcMYBA1* was isolated from blueberry fruit tissues and shown to be highly expressed in the fruit skin during ripening, when anthocyanin biosynthesis occurs (Plunkett et al. 2018). Furthermore, it was able to complement a defined SG6 MYB mutant (*Antirrhinum, rosea^dorsea^*) and induce anthocyanin accumulation when infiltrated into *Nicotiana benthamiana* leaves. *MYBA1* transgenes activated the promoters of several flavonoid genes, including *UFGT*, a critical biosynthesis step regulated specifically for anthocyanins in *Vaccinium*. Subsequently, SG6 MYBs from other *Vaccinium* species were identified and characterized. In bilberry, *VmMYBA1* is associated with ripening-associated anthocyanin accumulation in fruit and is downregulated in 2 wild mutants that produce albino fruit (Karppinen et al. 2021). Furthermore, treating mature green bilberry or blueberry fruit with abscisic acid rapidly accelerated ripening and anthocyanin accumulation (Karppinen et al. 2018; Oh et al. 2018) and rapidly induced *VmMYBA1* expression in bilberry (Karppinen et al. 2021). A *MYBA1* orthologue from wufanshu (*V. bracteatum*) has been characterized with even stronger anthocyanin biosynthesis activation properties than blueberry VcMYBA (Zhang et al. 2021). Analysis of the recent genome assemblies for tetraploid highbush blueberry (Colle et al. 2019), cranberry (Diaz-Garcia et al. 2021), and bilberry found this locus is complex, containing numerous *MYBA* copies, pseudogenes, and repeat elements (Wu et al. 2021). The location of the *MYBA* locus is syntenic between cranberry, blueberry, and bilberry, but considerable variation in the locus structure exists between species, suggesting gene duplication events have occurred since species divergence. These gene copies can be separated into 2 clades, “*MYBA1*” and “*MYBA2*” (Wu et al. 2021), suggesting existence of 2 ancestral genes with different promoter sequences that determine pigmentation patterns. In species studied to date, the *MYBA1* genes are expressed in fruit during ripening, while *MYBA2* copies may contribute to regulating pigmentation in other tissues, for example, light-regulated pigmentation in vegetative or floral tissues (Karppinen et al. 2021). The identification of the *MYBA* genes in *Vaccinium* species raises questions of how anthocyanin regulation is balanced with the production of other types of flavonoids during fruit development and how the regulatory mechanisms might overlap.

### PA regulation

In *Vaccinium* species PAs are a major class of flavonoids that accumulate throughout vegetative and reproductive tissues. Recent studies in bilberry and blueberry have identified several distinct MYB classes and gene families associated with PA regulation (Karppinen et al. 2021; Lafferty et al. 2022b). In bilberry this includes at least 5 *SG5/MYBPA2* genes, 2 *MYBPA1* genes, 2 *MYB5* genes, and a *PAR/MYBPA3* gene (Karppinen et al. 2021). *MYB5* contributes to seedcoat formation and seed mucilage in Arabidopsis and *M. truncatula* (Gonzalez et al. 2009; Li et al. 2009; Liu et al. 2014) and vacuole acidification in petunia and citrus (Quattrocchio et al. 2006; Zhang et al. 2019). *MYB5* expression can enhance PA accumulation in *M. truncatula*, petunia, and tobacco (Deluc et al. 2008; Cavallini et al. 2014) but seems to target a smaller subset of PA biosynthetic genes than SG5 MYBs (Liu et al. 2014). Liu et al (2014) showed that MBW complexes containing MtMYB14 (SG5) and MtMYB5 exist and act synergistically. The *PAR/MYBPA3* gene also contributes to PA regulation in *M. truncatula* (Verdier et al. 2012), and the bilberry homologue induced production of the PA precursor flavan-3-ol (gallocatechin) monomers when expressed in tobacco (Karppinen et al. 2021). The number of classes of MYBs involved in PA regulation and the lack of mutants for these (natural or induced) in grape or *Vaccinium* makes it difficult to establish the contribution of each regulator type. There is clearly some redundancy between these proteins for some target genes (e.g. MYBPA1 and MYBPA2 both regulate *DFR* and *ANR*), but they are not functionally equivalent, suggesting additional target genes for transport or polymerisation.

### Why so many MYBs?

Despite studies in grape, persimmon (*Diospyros kaki*), and poplar (*Populus* species) (Bogs et al. 2007; Akagi et al. 2010; James et al. 2017), the role of MYBPA1 proteins for flavonoid regulation has been unclear because their gene expression and target genes are associated with both PA and anthocyanin biosynthesis. However, recent evidence from *Vaccinium* helps to clarify their role as dual regulators, contributing to both pathways, reconciling findings from other fruit species. Silencing *MYBPA1* in bilberry resulted in a visible loss of anthocyanins and reduced expression of *CHS*, *F3ʹ5ʹH*, *ANS*, and *LAR1b*, demonstrating that MYBPA1 makes an essential contribution to anthocyanin regulation (Karppinen et al. 2021). Blueberry VcMYBPA1.1 could not induce anthocyanin or PA accumulation in tobacco leaves alone, but a modified version fused to a strong transcriptional repressor (MYBPA1.1-CREST) was a potent inhibitor when coexpressed with VcMYBA1 (of anthocyanin) or VcMYBPA2.2/VcMYBPA2.3 (of PAs). Promoter activation assays again showed MYBPA1.1 had strong activation activity against the promoters of *CHS*, *F3ʹ5ʹH*, and *ANR*, moderate activity against the *DFR* and *ANS*, but none against *UFGT* or the *MATE8* anthocyanin transporter (Lafferty et al. 2022a). These findings support a role for regulating key steps within the flavonoid pathway despite lacking the ability to regulate all the steps required for metabolite production and transport.

How is the activity of multiple MYBs coordinated for metabolite production? The answer lies in hierarchy: some TFs activate others. Hierarchical regulation of MYB repressor and bHLH2 genes is well established for anthocyanin and PA regulation (Albert et al. 2014), but regulation of MYB activators by MBW complexes is a recent addition. It was recently shown that *MYBPA1* genes are directly regulated by MBW complexes containing MYBPA2 or MYBA proteins (Lafferty et al. 2022b) (Fig. 1D). This key finding reconciles the expression profiles of *MYBPA1* in grape and *Vaccinium* species, with peak expression corresponding with PA regulation in immature fruit and a second peak when anthocyanin occurs (Bogs et al. 2007) (Fig. 1C). It also explains why grape hairy root cultures overexpressing *VvMYBPA2* have increased *VvMYBPA1* expression (Terrier et al. 2009). In apple, overexpressing *MdMYBPA1* enhances PA accumulation, and MdMYBPA2 and MdbHLH33 were shown to activate *MdMYBPA1* promoter (Wang et al. 2018). Anthocyanin accumulation was also enhanced by *MdMYBPA1* overexpression with low temperature when *MdMYB10* (SG6 MYB) was induced (Wang et al. 2018), suggesting that cooperative regulation of core flavonoid genes by MYBPA1 proteins with pathway specific MYBs is common in fruit.

SG5 MYBs may also contribute to anthocyanin regulation and modification. A recent study in strawberry characterized FaMYB123 (SG5) as a regulator of anthocyanin biosynthesis genes, including *ANS*, and genes for flavonoid decoration and acylation (glycosyl- and malonyl-transferases) but not those for PA biosynthesis (Martínez-Rivas *et al*. 2023). The activity was not redundant with the primary anthocyanin regulator, FaMYB10 (SG6), suggesting these also cooperatively act to regulate the production of more specialized anthocyanin types during fruit ripening. In blueberry, acylated anthocyanin species are restricted to late stages of fruit development when acyl transferases are expressed (Günther et al. 2020). It is not clear how these are regulated, but the lack of these more decorated forms of anthocyanins in vegetative tissues (Plunkett et al 2018) suggests additional regulators may be involved.

### Regulation of F3′5′H—a special target?

The presence of flavonoids with 3ʹ4ʹ5ʹ hydroxylation (e.g. delphinidin-based anthocyanins, myricetin-based flavonols) is sporadic throughout angiosperm families, with *F3ʹ5ʹH* thought to have evolved numerous times from ancestral *F3ʹH* genes (Seitz et al. 2015). This may be reflected by differences in regulation of *F3ʹ5ʹH* between groups of plants. For example, within the Solanaceae, *F3ʹ5ʹH* genes are regulated by SG6 MYBs (MBW) (Albert et al. 2014; Butelli et al. 2021), whereas in other plants activity is restricted to particular tissues, developmental stages, or conditions. In grape, differential regulation of *F3ʹ5ʹH* genes occurs between different SG6 MYBs: VviMYBA1 strongly activates several *F3ʹ5ʹH* genes, but VviMYBA6 and VviMYBA7 have weak to no activity. This results in different anthocyanin profiles between berries (higher delphinidin:cyanidin ratio) and vegetative tissues because these MYBs have distinct spatial and temporal expression patterns, *VviMYBA1* is the key determinant of fruit pigmentation, and *VviMYBA6/7* regulate anthocyanin in vegetative tissues (Matus et al. 2017). In blueberry, vegetative tissues accumulate only simple cyanidin-glycosides instead of the complex mixtures of cyanidin, delphinidin, petunidin, and malvidin-based anthocyanins present in fruit (Plunkett et al. 2018). This suggests a lack of F3ʹ5ʹH activity that may be even more restricted than in grape. However, it remains unclear how this difference in anthocyanin profiles arises, given different observations and data. Overexpression of *VcMYBA1* in blueberry plants resulted in red vegetative pigmentation, suggesting cyanidin accumulation (Lafferty et al. 2022a). This is somewhat surprising, given these plants express *MYBPA1*, *MYBPA2* in leaves, associated with PA accumulation, in addition to the *MYBA1* transgene (Lafferty et al. 2022a)—3 coexpressed MYBs that were each shown to activate the blueberry F3ʹ5ʹH in promoter activation assays (Lafferty et al. 2022b). And finally, cranberry has *F3ʹ5ʹH* activity associated with flavonol production during early stages of fruit development but lacks delphinidin-based anthocyanins (Vvedenskaya et al. 2004). Understanding how this occurs is of interest, given the role of F3ʹ5ʹH for generating diverse flavonoid compounds with altered properties, including color, stability, and health attributes. It is possible that distinct *F3ʹ5ʹH* genes are differentially regulated by flavonol versus anthocyanin MYBs, or perhaps it is regulated by a distinct regulator, as occurs in kiwifruit (*Actinidia* spp.) (Peng et al. 2019).

### Spatial and temporal patterning

Within *Vaccinium*, many species have anthocyanin restricted to the berry skin/peel, but there are notable red-fleshed exceptions. The best characterized is bilberry, but the red-flesh trait is shared with other members within *Vaccinium* section Myrtillus and sporadically arises within other parts of the genus (e.g. deerberry, *V. staminium*). These differences are exquisitely captured by mass-spectrometry imaging (Dare et al. 2022), which showed the spatial distribution of flavonoids as they are produced at different developmental stages. Most striking is the advance of anthocyanin patterning in line with maturity, with a strong correlation with sugars and, ultimately, how all this apparently coordinated metabolite production can be defined by chemical signatures that progress from the skin into the flesh.

How is this patterning established? In other species, altered expression of the SG6 MYB genes are central to generating new pigmentation patterns (reviewed in Albert et al. 2022). This can involve *cis* regulatory changes, such as promoter variants (e.g. apple; Espley et al. 2009) or transposon activation/enhancement (e.g. strawberry; Castillejo et al. 2020), or *trans* changes, altering the expression of upstream regulators of the *MYB* gene (e.g. red-flesh peach; Zhou et al. 2015; Hara-Kitagawa et al. 2020). In blueberry, *MYBA1* transcripts are highly abundant in the skin tissues at ripening and present in flesh tissues in trace amounts, and in bilberry, *MYBA1* transcripts are also expressed in the flesh, although still less than in skin (Lafferty et al. 2022a). This argues against red-flesh in bilberry being a loss of anthocyanin repressors, such as SG4 R2R3-MYBs, R3-MYB repressors, or SPLs, which antagonize the activity of MBW complexes (Aharoni et al. 2001; Gou et al. 2011; Albert et al. 2014; Cao et al. 2017). It is unknown if the genetic basis of red flesh in bilberry is linked to the *MYBA* locus itself or to another region, because it is not polymorphic for flesh color, preventing QTL mapping. Insights may come from inter-specific hybrids between deerberry and blueberry, which show that red flesh from deerberry is a dominant (or semi-dominant) trait (Lyrene 2021). If mapping populations can be generated, this would help address the basis of this trait in deerberry, which may be a shared mechanism in other red-fleshed *Vaccinium* species.

Small-RNAs are increasingly recognized for their roles regulating the expression of key TFs that control flavonoid metabolism. This includes *miR828* and *miR858*, which target *SG5 R2R3-MYB* and *MYBPA1* genes involved in PA regulation in many plants and commonly have an inverse expression pattern to the transcript abundance for the target *MYB* gene in fruit (e.g. apple, kiwifruit, persimmon) (Li et al. 2019; Yang et al. 2020; Zhang et al. 2022). A similar pattern occurs in blueberry, with *SG5/MYBPA2* transcript accumulation inverse to *miR858b* during blueberry fruit development, with degraded transcript detected at late stages when intact mRNAs are not abundant (Li et al. 2018). The pattern of PA accumulation in blueberry (and other fruits) closely follows the expression profile of *SG5/MYBPA2* genes (Li et al. 2018; Lafferty et al. 2022a); thus, miRNA regulation may determine the temporal expression pattern of *MYBPA2* genes during berry development.

### Genetic variation

A pangenome for *Vaccinium* was recently developed (Yocca 2022). A single reference genome sequence does not contain all genes within a species because genes may be present in every individual (core) or absent in at least one individual (auxiliary; Golicz et al. 2020). The *Vaccinium* pangenome was mined to assess gene presence across dozens of blueberry genotypes (Supplemental Fig. S1). Although most flavonoid biosynthesis genes and transcription factors are retained across all genotypes, some are missing in a few genotypes and others are only present in a single genome. Variation in retention of these genes may lead to cross-accession differences in anthocyanin accumulation or structure, which must be accounted for in future breeding decisions.

## Future directions

The recent surge in genomic resources and research has driven progress in understanding the production and regulation of the key flavonoids in *Vaccinium*. This now extends to functional data on the transcriptional regulation of the flavonoid pathway and the development of genetic markers. It also emphasizes the need to match resources across the range of *Vaccinium* species, allowing us to understand how, among other quality traits, breeders can develop improved cultivars and exploit the potential flavonoid-derived health benefits. Clearly, there is need for improved genetic resources in other *Vaccinium* species to help drive the breeding and selection of key traits, including the flavonoids that are so intrinsically linked with health properties. This need extends to the establishment of common gardens with sufficient germplasm resources for GxE-type experiments. Finally, the construction of *Vaccinium* pangenomes will help our understanding of wild relatives and the potential for introgression of valuable traits for further crop improvement.

The rich diversity of flavonoids in *Vaccinium* species, coupled with both the newly acquired and developing genetic resources, will provide new tools to understand flavonoid production, which can be applied across a range of fruit crops. These tools can build on those developed in grape, strawberry, apple, and other crops to answer questions such as what governs the pathway flux toward di- or trihydroxylated anthocyanins or the decoration and acylation of flavonoids as well as the factors that control flavonoid accumulation in different fruit tissues (flesh and skin).

## Supplementary Material

kiad250_Supplementary_Data

## Supplementary data

**Supplemental Fig. S1**. Presence-absence variation (PAV) of anthocyanin biosynthesis genes across northern highbush (NHB; **A**) and southern highbush (SHB; **B**) blueberry genotypes. Light blue indicates the gene is both syntenic and present.

**Supplemental Table S1**. Summary of flavonoid biosynthetic gene predictions from *Vaccinium* species and Arabidopsis, apple, strawberry, and grape. Predicted peptide sequences of Vitis vinifera PN40024 v4.1, Actinidia chinensis Red5 v1.0, Malus domestica v1.1, Fragaria vesca v4.0.a2, Vaccinium caesariense W85-20 v2.0, Vaccinium myrtillus isolate NK2018 v1.0, and Vaccinium macrocarpon cv Stevens v1.0 were retrieved. KIPEs v3.2.2 was applied with the flavonoid bait sequence collection v3.0 to identify the anthocyanin biosynthesis genes in these datasets. Proteins involved in anthocyanin decoration and transport were additionally checked through phylogenetic trees based on MAFFT v7 alignments and constructed with FastTree v2.1 (Price et al. 2010; Katoh and Standley 2013).

**Supplemental Table S2**. Summary of QTL for chlorogenic acid and anthocyanins concentration, anthocyanin composition mapped in blueberry genetic studies. Data were extracted from Mengist et al. (2022b) and Montanari et al. (2022).

**Supplemental Table S3**. Summary of quantitative trait loci for TAcy, PAC, color variation, and color intensity mapped in cranberry genetic studies. Data were extracted from Diaz-Garcia et al. (2018) and Diaz-Garcia et al. (2021).
