# Supplementary material for: Vaccinium as a comparative system for understanding of complex flavonoid accumulation profiles and regulation in fruit
Source: Plant Physiol. 2023 May 2;192(3):1696–710. doi: 10.1093/plphys/kiad250 (PMC10315322; doi:10.1093/plphys/kiad250)
Supplement: kiad250_Supplementary_Data [file kiad250_supplementary_data.zip › Supplementary Figure 1.pdf]

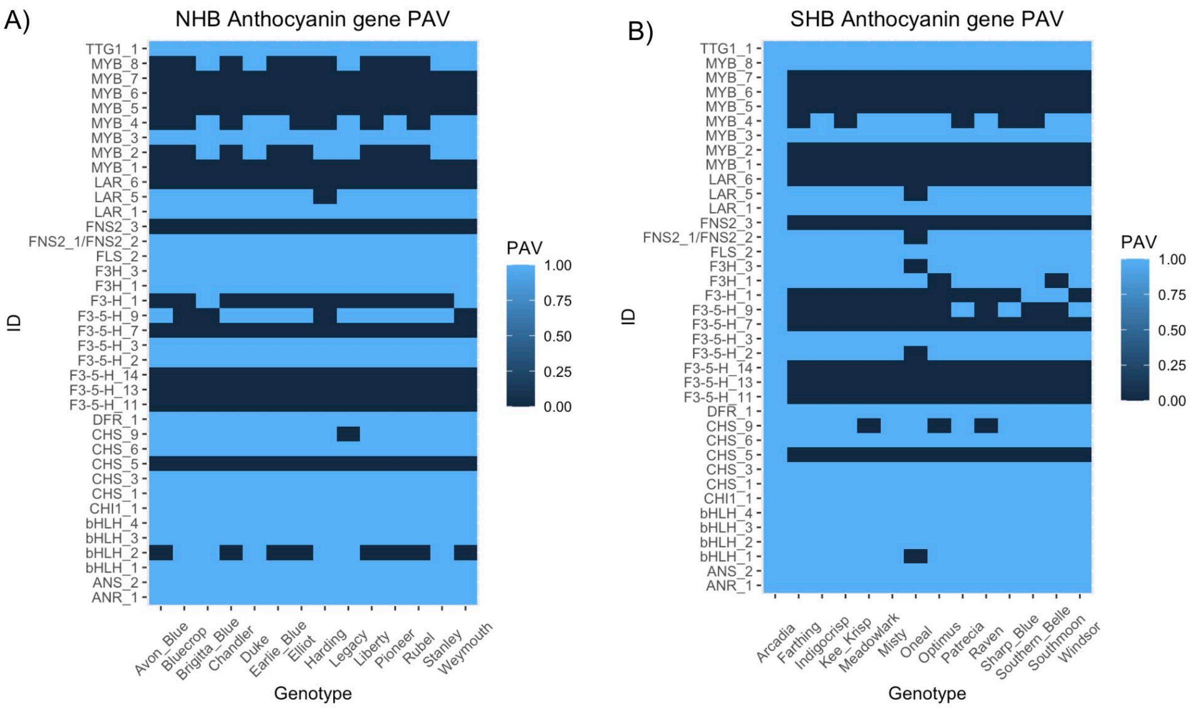

Supplementary Figure 1. Presence-absence variation (PAV) of anthocyanin biosynthesis genes across Northern Highbush (NHB; A) and Southern Highbush (SHB; B) blueberry genotypes. Light blue indicates the gene is both syntenic and present.
